# Use of Optical Redox Imaging to Quantify Alveolar Macrophage Redox State in Infants: Proof of Concept Experiments in a Murine Model and Human Tracheal Aspirates Samples

**DOI:** 10.3390/antiox13050546

**Published:** 2024-04-29

**Authors:** He N. Xu, Diego Gonzalves, Jonathan H. Hoffman, Joseph A. Baur, Lin Z. Li, Erik A. Jensen

**Affiliations:** 1 Britton Chance Laboratory of Redox Imaging, Department of Radiology, Perelman School of Medicine, University of Pennsylvania, Philadelphia, PA 19104, USA; jhh286@cornell.edu (J.H.H.); linli@pennmedicine.upenn.edu (L.Z.L.); 2 Department of Surgery, Perelman School of Medicine, University of Pennsylvania, Philadelphia, PA 19104, USA; dgonzalv@sas.upenn.edu; 3 Department of Physiology, and Institute for Diabetes, Obesity, and Metabolism, Perelman School of Medicine, University of Pennsylvania, Philadelphia, PA 19104, USA; baur@pennmedicine.upenn.edu; 4 Department of Pediatrics, Children’s Hospital of Philadelphia and Perelman School of Medicine, University of Pennsylvania, Philadelphia, PA 19104, USA; jensene@chop.edu

**Keywords:** nicotinamide adenine dinucleotide (NAD^+^ and NADH), autofluorescence fluorescence imaging, mitochondrial redox state, mitochondrial ROS, alveolar/lung macrophages, preterm infants, bronchopulmonary dysplasia (BPD), gestational age, mean airway pressure

## Abstract

Emerging data indicate that lung macrophages (LM) may provide a novel biomarker to classify disease endotypes in bronchopulmonary dysplasia (BPD), a form of infant chronic lung disease, and that augmentation of the LM phenotype may be a potential therapeutic target. To contribute to this area of research, we first used Optical Redox Imaging (ORI) to characterize the responses to H_2_O_2-_induced oxidative stress and caffeine treatment in an in vitro model of mouse alveolar macrophages (AM). H_2_O_2_ caused a dose-dependent decrease in NADH and an increase in FAD-containing flavoproteins (Fp) and the redox ratio Fp/(NADH + Fp). Caffeine treatment did not affect Fp but significantly decreased NADH with doses of ≥50 µM, and 1000 µM caffeine treatment significantly increased the redox ratio and decreased the baseline level of mitochondrial ROS (reactive oxygen species). However, regardless of whether AM were pretreated with caffeine or not, the mitochondrial ROS levels increased to similar levels after H_2_O_2_ challenge. We then investigated the feasibility of utilizing ORI to examine macrophage redox status in tracheal aspirate (TA) samples obtained from premature infants receiving invasive ventilation. We observed significant heterogeneity in NADH, Fp, Fp/(NADH + Fp), and mitochondrial ROS of the TA macrophages. We found a possible positive correlation between gestational age and NADH and a negative correlation between mean airway pressure and NADH that provides hypotheses for future testing. Our study demonstrates that ORI is a feasible technique to characterize macrophage redox state in infant TA samples and supports further use of this method to investigate lung macrophage-mediated disease endotypes in BPD.

## 1. Introduction

Approximately one of every nine live-born infants is delivered prematurely in the U.S [1]. These premature infants are at risk of developing bronchopulmonary dysplasia (BPD). BPD is a severe chronic lung disease that develops over the first months of age after premature birth and is diagnosed and severity graded (grade 1–3) by the respiratory support administered at 36 weeks postmenstrual age (PMA = gestational age [GA] at birth + chronologic age) [2,3,4,5]. BPD affects ~50% of extremely preterm infants (those born with GA ≤ 28 weeks), is the 2nd most common chronic pediatric lung illness after asthma, is a strong predictor of life-long health impairments, and accounts for more than USD 2.4 billion per year in US healthcare costs [6,7,8]. Rates of BPD have not declined over the past 30 years and few therapies prevent or treat this disease [9,10]. Available data indicate that the pathobiology responsible for BPD development and exacerbation is heterogeneous and marked by varying levels of lung and pulmonary vascular maldevelopment, in utero and postnatal inflammation, hyper/hypoxic lung injury, infection-mediated lung damage, and abnormal host repair. Motivated by these findings, investigators have emphasized the identification of disease phenotypes (shared clinical characteristics) and endotypes (shared underlying biology) in BPD as a key area of research in hopes of discovering more effective, pathology-specific treatments [4,11].

Both prenatal (e.g., genetic/epigenetic risk factors, intrauterine hypoxia, infection, nutrition) and early postnatal risk factors (e.g., mechanical ventilation and oxygen supplementation) underlie the development of BPD [12]. Dysregulated metabolism involving glucose, lipids, and amino acids has been identified in various cell types, including epithelial cells, fibroblasts, airway smooth muscle cells, and endothelial cells in infants with BPD [13]. Often the acuity of lung disease correlates with the observed severity of these metabolic abnormalities [13]. The alveolar macrophage (AM) is the predominant inflammatory cell type within the alveolar structure in BPD and their activation and function are closely associated with BPD development [14]. Transcriptional profiling of tracheal aspirate (TA) macrophages predicts inflammation-mediated lung disease in preterm infants and higher expression of inflammatory mediators can be detected as early as the first day after birth in newborns who eventually develop BPD [15]. Hydrogen peroxide (H_2_O_2_) released by AM collected from bronchoalveolar lavage has been shown to be higher in preterm infants with BPD than in those without parenchymal lung disease. Moreover, corticosteroid therapy alters the activation status of AM preceding recovery of lung function in established BPD [16], indicating that lung macrophages may be a sensitive and early indicator of lung disease trajectory. Macrophages isolated from tracheal aspirate samples obtained during routine clinical suctioning of the endotracheal tube in intubated infants are easily obtained and are a highly relevant innate immune cell type in BPD pathogenesis and recovery [15,17,18]. Therefore, studying the metabolic state of macrophages from TA samples holds promise to help characterize BPD endotypes and support the individualization of therapeutic interventions. 

Macrophages assume a spectrum of phenotypes between pro-inflammatory (M_1_) and anti-inflammatory (M_2_) extremes [15,19,20] with M_1_ characterized primarily by glycolysis and M_2_ by oxidative phosphorylation as their metabolic signatures [21]. Phenotypic and functional changes in macrophages require significant metabolic reprogramming [21,22,23,24,25,26] that is critically supported by altered mitochondrial redox metabolism. Nicotinamide adenine dinucleotide (NADH and its oxidized form NAD^+^) together with flavin adenine dinucleotide (FAD and its reduced form FADH_2_) are essential coenzymes that critically support ATP production and maintain mitochondrial redox homeostasis by binding to the relevant proteins. The fluorescence signals of NADH and FAD are sensitive to dynamic changes in intracellular metabolism in macrophages in tissue repair [27] (only NADH and FAD have intrinsic fluorescence whereas NAD^+^ and FADH_2_ do not fluoresce). Optical redox imaging (ORI) is a label-free technique that records the intrinsic fluorescence intensities of NADH and FAD-containing flavoproteins (Fp), both of which are primarily found in mitochondria [28,29], and provide a measure of cellular metabolism and the mitochondrial redox state [28,30,31]. The mitochondrial redox state can be quantified by the (optical) redox ratio: Fp/(NADH + Fp) [32]. Mitochondrial redox metabolism regulates the generation of reactive oxygen species (ROS) during respiration. In addition, the redox ratio and the concentrations of NADH and Fp can also reflect and quantify fluctuations in cellular and mitochondrial ROS [33,34,35,36,37]. Thus, characterizing the mitochondrial redox status of macrophages using ORI can indicate their functional activity and may serve as a useful biomarker in infant lung disease.

Previously, we showed that the mitochondrial redox state of ex vivo alveolar macrophages from transgenic mice exposed to ozone (a strong oxidant) is surfactant protein-A2 and sex-dependent [35,36]. In human infants, surfactant deficiency and oxidative injury from hyperoxia predispose to BPD [38], and male infants are at higher risk of developing BPD than female infants. Thus, our ex vivo data combined with these clinically observed phenomena support the hypothesis that ORI of lung macrophages may help elucidate mechanisms that underly specific genetic and early postnatal risk factors associated with the development of BPD. 

Here we report method development for the novel application of ORI to study tracheal aspirates from infants receiving invasive ventilation in a clinical setting. Since oxidative stress is a major postnatal risk factor for BPD and caffeine is currently one of the few drugs shown to significantly reduce the risk of BPD [39,40], we first examined in an in vitro mouse AM model how oxidative stress and caffeine affect the mitochondrial redox status of macrophages. We then describe the application of ORI to investigate the mitochondrial redox status of TA macrophages collected from 17 ventilator-dependent infants. 

## 2. Materials and Methods

### 2.1. Alveolar Macrophage Cell Line Culture and Treatment

Mouse alveolar macrophage cell line (MH-S) purchased from ATCC (Manassas, VA, USA) were cultured in 10% FBS supplemented RPMI1640 (with L-glutamine) at 37 °C and 5% CO_2_. As per our pre-defined experimental designs, the cells were either acutely treated with increasing concentration of H_2_O_2_ (in a serial addition manner, ranging from 0 to 3.6 mM) with each addition lasting 2 min followed by imaging, or caffeine treatment for 16 h followed by imaging, or caffeine pretreatment for 16 h followed by ~30 min of H_2_O_2_ exposure then imaging. To perform the metabolic modulation experiment to detect NADH dynamic range, two dishes of cells were prepared, one with 900 μM H_2_O_2_ addition, and one without. To obtain the oxidized extreme, these dishes were first treated with carbonyl cyanide p-trifluoro-methoxyphenyl hydrazone (FCCP, 0.5 µM) and imaged within 5 min. The dishes were then treated with a mixture of mitochondrial inhibitors rotenone and antimycin A (ROTAA, ROT = 1 μM and AA = 1.25 μg/mL) and imaged again within 5 min to obtain the reduced extreme. All chemicals were purchased from Sigma-Aldrich (Saint Louis, MO, USA). 

### 2.2. TA Sample Collection and Processing

TA samples were collected anonymously using a waiver of informed consent in the Newborn/Infant Intensive Care Unit at the Children’s Hospital of Philadelphia under IRB protocol 21-019057 approved on 22 July 2021. All TA samples were collected by nursing or respiratory staff at times of routine, clinically indicated endotracheal suctioning. These samples are typically discarded but were collected and utilized for this research study. Each infant was only sampled once for this analysis. To maintain participant anonymity and enable performance of this method development and proof of concept study under a waiver of informed consent, only a small amount of clinical information was recorded on each study subject. 

Sample collection was performed by advancing an in-line suction catheter just proximal to the end of the in-situ endotracheal tube followed by aspiration of tracheal secretions into a sterile Lukens trap. EDTA (final concentration 0.5 mM) was added to the trap within 30 min of collection to prevent adherence of macrophages to the trap surface. The samples were then placed on ice and transported to the lab for processing and subsequent imaging. 

To remove EDTA, the TA fluid was transferred to a 15 mL centrifuge tube, 9 mL of PBS was added to each tube, and centrifugation was performed at 1000 rpm for 6 min. To release the cells trapped in phlegm, the mucus plugs in the samples were transferred to a 6-well plate and treated with dithiothreitol (DTT, final concentration 6.5 mM). The plate was put on ice and placed on a rocker for 20 min. The remaining portion of the TA sample contained primarily loose cells, which were transferred to the glass-bottom dishes and incubated in RPMI1640 (with L-glutamine) + 10% fetal bovine serum (FBS) at 37 °C and 5% CO_2_. The loose cells are hereafter referred to as group A. For this proof-of-concept study, we tested a second approach to isolate lung macrophages, hereafter referred to as group B cells. For this approach, after 20 min of DTT treatment, the samples were filtered with 50 µm cell strainers and spun down to remove DTT followed by 1 rinse with 10 mL of PBS. The DTT-released cells (i.e., group B) were then transferred to the glass-bottom dishes and incubated with RPMI1640 + 10% FBS at 37 °C and 5% CO_2_. Incubation was conducted for ~3.5 h before imaging.

**Figure 1:**
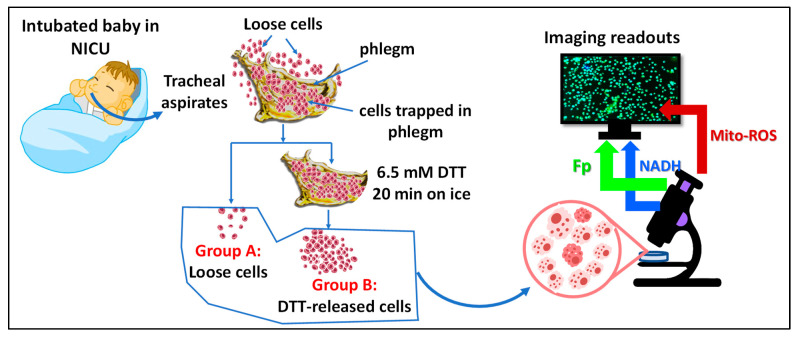
The experiment schematics and tracheal aspirate (TA) sample processing.

After ~3.5 h of incubation after seeding, cells in group A and group B were first subjected to ORI in an interleaved manner. After ORI, a red fluorescence dye (MitoSOX^TM^ red, Thermo Fisher Scientific, Waltham, MA USA) was added to the dishes to quantify mitochondrial ROS levels. Figure 1 provides a schematic of sequential steps used for imaging the mitochondrial redox status of the TA samples.

### 2.3. Optical Redox Imaging and Data Processing

To minimize background fluorescence, the complete medium was first aspirated out and the dishes were rinsed twice with PBS and then cultured in live cell imaging solution (Life Technologies, Carlsbad, CA, USA) supplemented with 11 mM glucose and 2 mM L-glutamine (LCIS^+^) for imaging [33,35,36]. The aspiration and rinsing process removed the nonadherent immune cells in the TA samples and left only the attached macrophages for imaging. 

The imaging and data processing details have been reported in our previous works [33,35,36,41]. Briefly, ORI was carried out with an inverted wide-field Zeiss fluorescence microscope equipped with a temperature chamber set at 37 °C (Axio Observer 7, ZEISS, Oberkochen, Germany) with a Plan-Apochromat 20×/0.8 M27 lens. The image resolution and size were 0.293 µm × 0.293 µm^2^/pixel and 562.56 µm × 356.29 µm/field of view (FOV), respectively. The intrinsic fluorescence of NADH and Fp were first imaged using the following filter sets: for NADH, excitation (Ex) 370–400 nm, emission (Em) 414–450 nm; for Fp, Ex 450–488 nm, Em 500–530 nm. After ORI, the dishes were administered with MitoSOX^TM^ Red with a final concentration of 2 µM for imaging mitochondrial superoxide in the live cells. The dishes were incubated at 37 °C for 10 min, followed by 2 rinses with PBS, then imaged with Ex 370–400 nm and Em 580–610 nm setting. For lipid peroxidation detection, the Cell-based Lipid Peroxidation Assay Kit (Abcam, Fremont, CA, USA, Cat. # ab243377) was used per the manufacturer’s instructions, where the green fluorescence was imaged with Ex 450–488 nm, Em 500–530 nm, and the red fluorescence was imaged with Ex 577–604 nm and Em 619–675 nm. The green and red fluorescence intensities were quantified, and their ratio was then calculated to obtain the lipid peroxidation index. To image mitochondrial dynamic network structure, MitoView^TM^ green (Biotium, Fremont, CA, USA) was used to stain mitochondria according to the manufacturer’s manual and imaged with a STELLARIS confocal microscope (Leica Microsystems, Inc., Deerfield, IL, USA)

Quantification of images was performed with a customized routine in MATLAB^®^ (MathWorks, Natick, MA, USA). Briefly, the cell-free background signals were subtracted from each raw image. The background-subtracted images were then thresholded at 7.5 signal-to-noise ratio for ORI images and 5 for MitoSOX images, respectively, where noise is defined as the standard deviation of the background signal. The redox ratio Fp/(NADH + Fp) images were generated pixel-by-pixel using NADH and Fp images after thresholding. The mean values of each of the redox indices (NADH, Fp, the redox ratio) or MitoSOX of each FOV were averaged to obtain the mean values representing a patient’s TA sample in the case of clinical samples or further averaged across culture dishes to obtain group means for the mouse AM cell line experiments unless otherwise indicated.

### 2.4. Statistics

Statistical analysis used Student’s t-test, ordinary one-way ANOVA with Dunnett’s multiple comparison correction, or simple linear regression using PRISM 9 (GraphPad Software, Boston, MA, USA). *p* < 0.05 is considered statistically significant. Significant differences are displayed as: *, *p* < 0.05; **, *p* < 0.01; ***, *p* < 0.001; and ****, *p* < 0.0001 in the reported figures.

## 3. Results

### 3.1. The Effects of H_2_O_2_ and Caffeine on Alveolar Macrophages In Vitro

#### 3.1.1. Acute H_2_O_2_ Effects

**Figure 2:**
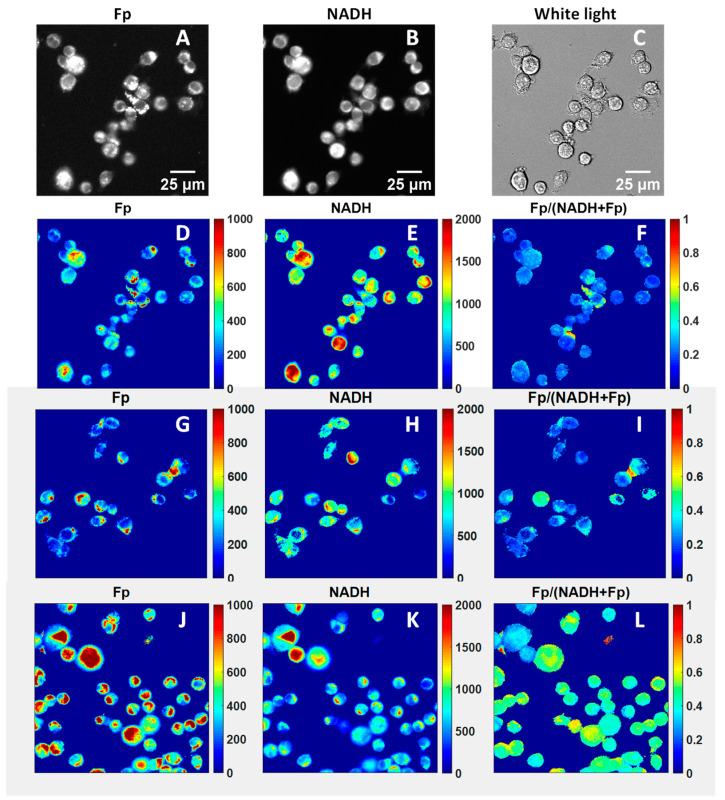
Representative images of mouse alveolar macrophages. Images (**A**–**C**) are raw images of AM under normal conditions. (**D**) and (**E**) are processed redox images corresponding to raw images (**A**) and (**B**), respectively. (**F**) is the corresponding redox ratio image. Images (**G**–**I**) and (**J**–**L**) are typical processed redox images of AM under 0.45 and 0.9 mM H_2_O_2_, respectively. The color bars indicate the pixel intensities or redox ratio (0–1) with red being more intense, i.e., the pixel being either higher NADH level or higher Fp level, or higher redox ratio, and blue less intense, i.e., the pixel being either lower NADH level, or lower Fp level, or lower redox ratio.

**Figure 3:**
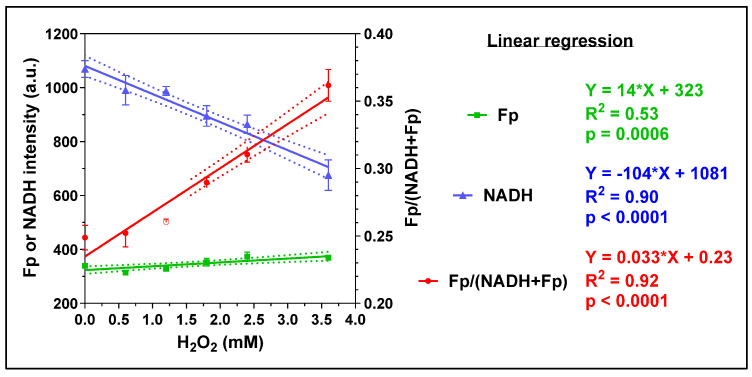
The immediate redox responses to acute exogenous H_2_O_2_ challenge at increasing concentrations, where the intensities of Fp and NADH are shown on the left *y*-axis and the redox ratio on the right *y*-axis. (mean ± SD, N = 3 dishes). Regression equation, goodness of fit (R^2^), and *p*-value corresponding to the regression slope are shown.

Exogenous H_2_O_2_ has been widely used as an oxidative stimulus to study the effects of intracellular reactive oxygen species (ROS) in a controlled manner. Mitochondria, as the central hub of cellular metabolism, critically support immune functions but are also a common target of ROS. We investigated the acute effects of exogenous H_2_O_2_ on AM using various imaging methods. Figure 2 displays the typical optical redox images of mouse AM. Compared to other cells, macrophages exhibit much higher resistance to oxidative stress. For example, under the treatment of 5 mM H_2_O_2_ for 24 h, they are able to maintain ~70% viability [42]. In our experiment, we progressively increased the H_2_O_2_ concentration from 0 to 3.6 mM by serially adding H_2_O_2_ to the AM culture. The cells were imaged two minutes after each addition. We observed linearly increasing Fp signals, linearly decreasing NADH, and linearly increasing redox ratio with increasing H_2_O_2_ dose (Figure 3), indicating that each progressive increase in H_2_O_2_ dose further shifted the mitochondria to a more oxidized state in a dose-dependent manner. 

**Figure 4:**
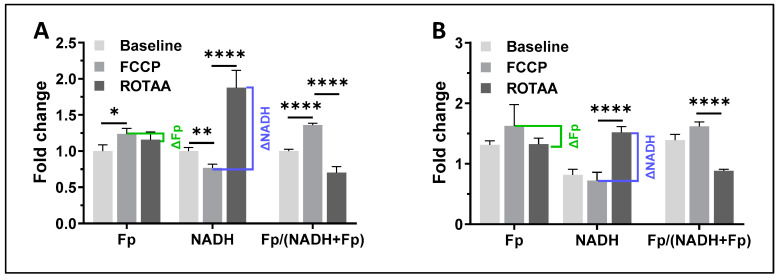
Redox titration results: (**A**) under normal condition (control); (**B**) under treatment with 0.9 mM H_2_O_2_ for 30 min., the baselines of the 3 redox indices were normalized to that of the normal condition (shown in (**A**)), respectively; Bars: mean ± SD, N ≥ 3 FOVs, the experiment was replicated with 0.45 mM H_2_O_2_. *, *p* < 0.05, **, *p* < 0.01, ****, *p* < 0.0001.

Since ROS-induced DNA damage is known to exhaust intracellular NAD^+^ due to the activation of NAD^+^-dependent DNA repair mechanisms [43,44,45,46] and here we observed an immediate decrease in NADH after each addition of H_2_O_2_, we suspected that ROS will also diminish the availability of NADH. This can be confirmed by observing a reduced dynamic range of NADH. To evaluate the NADH dynamic range, we modulated cellular metabolic states and imaged the redox responses [33,47,48]. We first applied FCCP to AM culture to uncouple mitochondrial oxidative phosphorylation, then added the mitochondrial inhibitors rotenone (ROT) plus antimycin A (AA), which inhibits complex I and III, respectively. Under complete uncoupling with FCCP, all NADH turns into NAD^+^ and a lower NADH signal is expected, which is the oxidized extreme. Conversely, under ROT and AA treatment, there is a buildup of NADH and an increased NADH signal is expected, which is the reduced extreme. The remaining signals are likely autofluorescence of other molecules. By calculating the difference in NADH between the two redox extremes, we obtained the dynamic ranges of NADH and Fp, denoted as ΔNADH and ΔFp, respectively. As anticipated, when the cells were challenged with 0.9 mM H_2_O_2_, we found a significantly lower ΔNADH (~28% decrease) and a significantly higher ΔFp (~276% increase) (Figure 4A,B). Significant decrease in ΔNADH and an increase in ΔFp were also observed after AM was treated with 0.45 mM H_2_O_2_, although with a less pronounced magnitude of change. 

**Figure 5:**
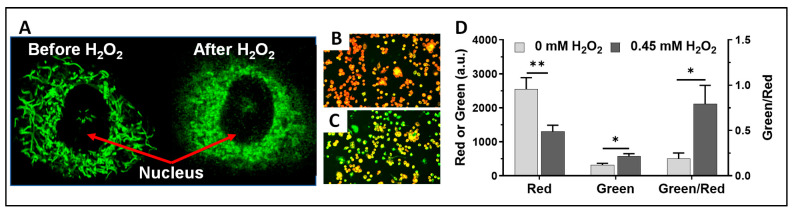
H_2_O_2_-induced changes in AM. (**A**) Mitochondria were stained with MitoView green (images were acquired with a Leica STELLARIS confocal microscope). Under normal control conditions, the alveolar macrophage showed a clear well-defined mitochondrial network structure, whereas the same cell after exposure to exogenous H_2_O_2_ (0.45 mM) showed a diffused mitochondrial network structure; (**B**,**C**) are representative overlay images of control (0 mM H_2_O_2_) and 0.45 mM H_2_O_2_ challenge conditions, respectively. Under control conditions (i.e., (**B**)), both red and green signals were recorded. Under 0.45 mM H_2_O_2_ challenge (i.e., (**C**)), both red and green signals were also recorded. The intensities of red and green fluorescence signals were adjusted to the same color ranges for the same channel for both control and 0.45 mM H_2_O_2_ challenge conditions. Visually, (**B**) has more red pixels and less green pixels, indicating less lipid peroxidation. (**C**) has more green pixels and less red pixels, indicating more lipid peroxidation. When red and green pixels co-localize, a yellow pixel appears. (**D**) Shows the quantification of the lipid peroxidation effects induced by exogenous 0.45 mM H_2_O_2_ where the intensities of green and red fluorescence are read on the left axis and their ratio on the right axis, N = 10 FOVs, and the error bars are standard errors; the experiment was replicated once with similar results. *, *p* < 0.05, **, *p* < 0.01.

To gain insights into how oxidative stress affects the mitochondrial structure, we stained the mitochondria of mouse AM with MitoView green fluorescence tracer and acquired the mitochondrial dynamic network image of a single cell. We then added 0.45 mM H_2_O_2_ to the culture and immediately imaged the same cell. As shown in Figure 5A, under normal conditions, the AM cell showed a well-defined mitochondrial dynamic network structure, whereas the same cell under H_2_O_2_ insult showed a diffused and ruptured mitochondrial network structure, indicating an immediate rupture (<1 min) of the mitochondrial dynamic network with 0.45 mM H_2_O_2_. Of note, this occurred at only half the concentration of H_2_O_2_ that we used to quantify the dynamic ranges of NADH and Fp shown in Figure 4.

Oxidative stress also significantly damages cell membranes. We used a ratiometric lipid peroxidation sensor to image the effects of oxidative stress on the AM membranes. Figure 5B,C are representative composite images of the lipid peroxidation sensor before and after exposing AM to 0.45 mM H_2_O_2_, respectively. The lipid peroxidation sensor localizes to the cell membranes and changes from red to green fluorescence in response to lipid peroxidation, providing a ratiometric indication of lipid peroxidation. Figure 4D shows lipid peroxidation quantification results, where there is a significant decrease in red fluorescence, an increase in green fluorescence, and a higher green/red fluorescence ratio due to H_2_O_2_. 

#### 3.1.2. Caffeine Effects

**Figure 6:**
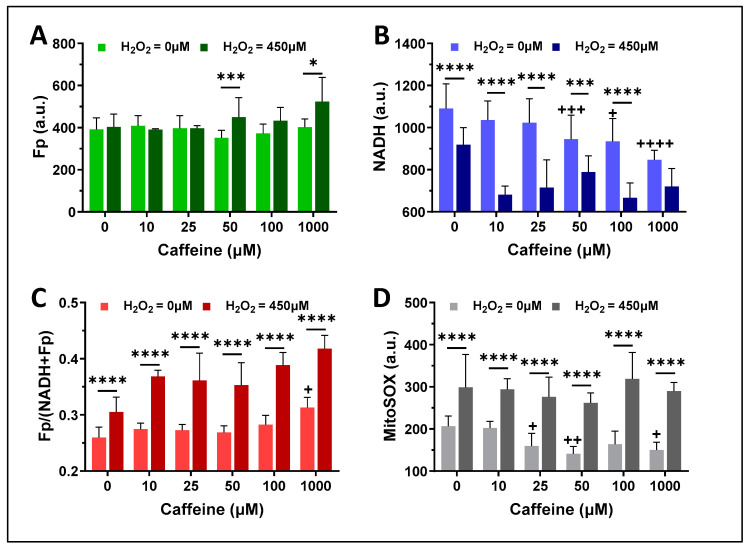
The redox effects of caffeine treatment alone (16 h, the bars with lighter colors) and the redox effects of H_2_O_2_ (450 µM, 30 min) on the AM pre-treated with caffeine at various doses for 16 h (the bars with darker colors). (**A**) The effects on Fp; (**B**) the effects on NADH; (**C**) The effects on the mitochondrial redox ratio; (**D**) The effects on the mitochondrial ROS. Bars: mean ± SD (N = 4 dishes), * or **^+^**, *p* < 0.05, **^++^**, *p* < 0.01, *** or **^+++^**, *p* < 0.001, **** or **^++++^**, *p* < 0.0001, where **^+^** or **^++^**, or **^++^**or**^+++^** or **^++++^** indicates the statistical comparisons against the control (no caffeine pretreatment or exposure to H_2_O_2_).

Using the same mouse AM cell line in vitro model system, we examined whether caffeine affected the mitochondrial redox status of mouse alveolar macrophages over a range of caffeine concentrations (0–1000 µM). As indicated with the lighter color bars in Figure 6A–C, after 16 h of caffeine exposure at various concentrations, Fp of AM (the light green bars in Figure 6A) showed no significant change, but NADH (the light blue bars in Figure 6B) began to decrease significantly starting at 50 µM of caffeine exposure and the redox ratio (the light red bars in Figure 6C) significantly increased when caffeine reached 1000 µM. The mitochondrial ROS levels (the light grey bars in Figure 6D) after 25–1000 µM caffeine treatment decreased significantly to below the baseline level (i.e., 0 µM). 

To test whether caffeine pretreatment may protect AM from acute oxidative stress, we first treated the AM culture for 16 h with caffeine at various doses (0–1000 µM), then challenged the caffeine-pre-treated AM culture with 450 µM H_2_O_2_. After a 30-minute exposure to 450 µM H_2_O_2_, in comparison with the respective ones without H_2_O_2_ exposure, there was a significant increase in Fp at caffeine doses of 50 µM and 1000 µM, a highly significant decrease in NADH at all caffeine doses except 1000 µM at which NADH decrease was not statistically significant compared to untreated control, and a highly significant increase in the redox ratio at all caffeine doses (Figure 6A–C). Furthermore, mitochondrial ROS levels were significantly increased compared to the respective ones without H_2_O_2_ exposure at all caffeine concentrations (Figure 6D). 

### 3.2. ORI of Infant Tracheal Aspirate Samples

**Figure 7:**
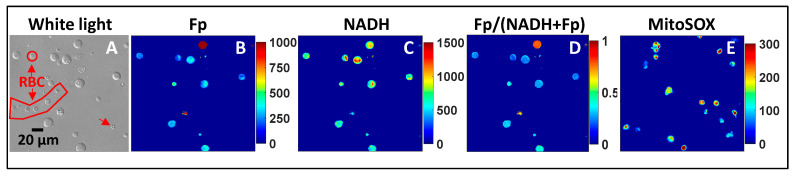
Typical white light and pseudo-colored redox and ROS images of a TA sample. (**A**) Shows the white light image of macrophages and a small number of red blood cells (RBCs) as indicated; (**B**–**D**) are the corresponding redox images with the color bars indicating their intensities or redox ratio (0–1). The RBCs do not have either Fp or NADH signals due to a lack of mitochondria; (**E**) is a typical mitochondrial ROS image (different FOV from that of the redox images).

Seventeen infants underwent tracheal aspirate collection. Their basic clinical characteristics are shown in Table 1. Figure 7 displays the typical white light images, the pseudo-colored redox images, and the mitochondrial ROS image of processed tracheal aspirate samples. Most of the attached cells were macrophages, although occasional red blood cells (RBCs, Figure 7A) from trace amounts of blood that remained in some TA samples werepresent. RBCs do not have Fp or NADH signals due to a lack of mitochondria (Figure 7B–D). We observed within-subject heterogeneity in macrophage Fp or NADH signal intensities and the corresponding redox ratio as shown by the color range in the redox images (Figure 7B–D). Figure 7E is a typical mitochondrial ROS image of the same TA sample (but different FOV), which also showed various ROS levels as indicated by the various colors. Note that some cells became detached and lost during MitoSOX staining and rinsing processes, preventing complete imaging of the full complement of cells that underwent redox imaging.

**Figure 8:**
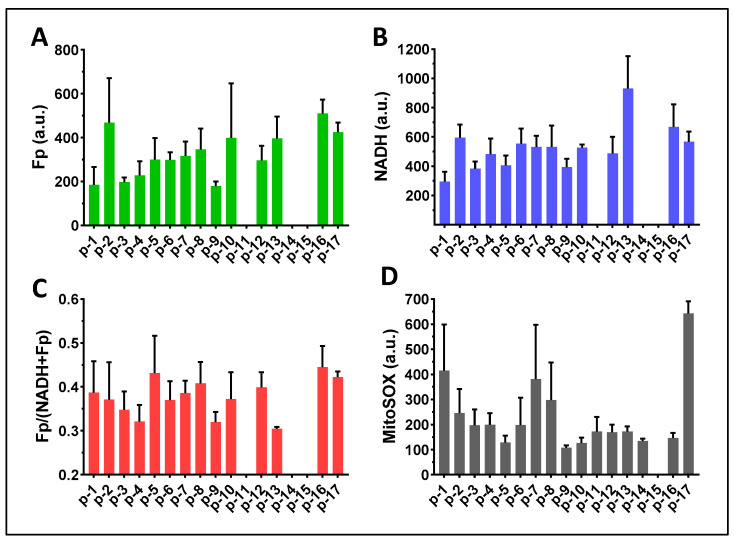
Image quantification of TA macrophages in group A (16 TA samples). (**A**) Global averaging quantification for Fp (14 TA samples); (**B**) Global averaging quantification for NADH (14 TA samples); (**C**) Global averaging quantification for the redox ratio (14 TA samples); (**D**) Global averaging quantification for the mitochondrial ROS (16 TA samples). The x-axes represent individual patients. Bars: mean ± SD, where SD is the standard deviation of ≥5 FOVs for the specific TA sample.

The loose cells (group A) and DTT-released cells (group B) were imaged alternately in the same imaging session. Figure 8 presents the global averaging quantification results for the cells of group A. Values for all imaging indices varied in this modestly sized cohort of predominately premature-born ventilator-dependent infants. The Fp intensity varied between 200–500 a.u.; the NADH intensity varied between 250–950 a.u.; the redox ratio varied between 0.3–0.45; the mitochondrial ROS level varied between 120–650 a.u. The large error bars representing the standard deviations indicate that the cell populations in each FOV were highly heterogeneous in terms of their redox status or mitochondrial ROS level. Of note, we were not able to collect loose cells from one TA sample. Additionally, two TA samples had abnormally high fluorescence backgrounds and were excluded from subsequent analyses. We were unable to determine whether these two patients were receiving therapeutics with high fluorescent properties as all TA samples were collected anonymously without corresponding drug treatment information.

**Figure 9:**
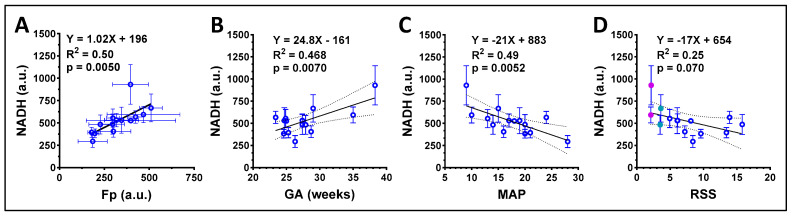
Correlations between the redox indices and the clinical parameters of group A macrophages, each circle represents a unique patient. (**A**) Fp positively correlated with NADH; (**B**) NADH positively correlated with gestational age (GA). (**C**) NADH negatively correlated with ventilator mean airway pressure (MAP). (**D**) NADH negatively correlated with respiratory severity score (RSS) with a borderline significance (*p* = 0.070), where for the two samples with the same RSS (magenta and green dots), higher NADH was observed in the infant with a higher GA. Regression equation, goodness of fit (R^2^), and *p*-value corresponding to the regression slope are shown.

Linear correlation analysis revealed a significant correlation between Fp and NADH (Figure 9A). Furthermore, NADH signals positively and significantly correlated with gestational age (GA) (R^2^ = 0.468, *p* = 0.0070, Figure 9B). We also found that there was a significant negative correlation between the NADH signal and the ventilator mean airway pressure (MAP) (R^2^ = 0.49, *p* = 0.0052, Figure 9C). In addition, as shown in Figure 9D, there was a marginally significant negative correlation between NADH and respiratory severity score (RSS, defined as the fraction of inspired oxygen multiplied by MAP). While two patients had the same RSS (magenta and green dots in Figure 9D), the one with higher NADH also had higher GA.

**Figure 10:**
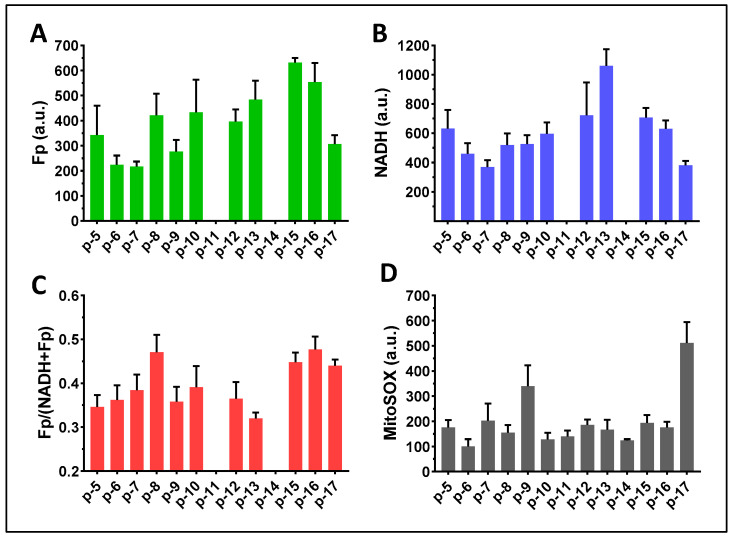
Image quantification of TA macrophages in group B where the cells were released from phlegm with DTT treatment. (**A**–**C**) Global averaging quantification of 11 TA samples for Fp, NADH, and the redox ratio, respectively; (**D**) Global averaging quantification for mitochondrial ROS (13 TA samples). Bars: mean ± SD, where SD is the standard deviation of ≥5 FOVs for the specific TA sample.

Figure 10 presents the global averaging quantification results for the DTT-released cells (group B). Note that we did not have DTT-released cells from four patients (p-1, p-2, p-3, p-4) as these four samples were our first batch of TA specimens collected for redox imaging, and the phlegm was not treated with DTT in these samples. Similar to those in group A, Fp intensity varied between 220 and 650 a.u.; NADH intensity varied between 350 and 1100 a.u.; the redox ratio varied between 0.3–0.48; the mitochondrial ROS level varied between 100 and 550 a.u.

**Figure 11:**
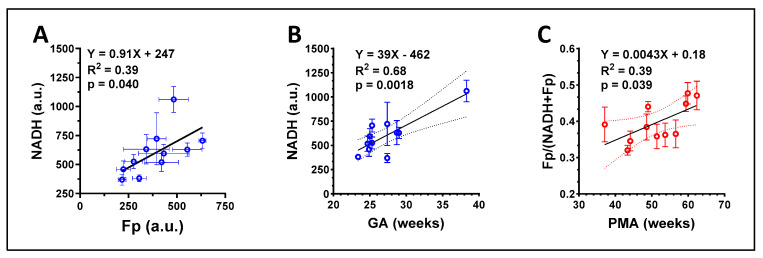
Correlations between the redox indices of group B and clinical parameters. (**A**) A significant positive correlation between Fp and NADH; (**B**) A significant positive correlation between the NADH level and subject gestational age; (**C**) A significant positive correlation between the redox ratio and the postmenstrual age (PMA) at sample collection. Regression equation, goodness of fit (R^2^), and *p*-value corresponding to the regression slope are shown.

Linear regression analyses performed on samples in group B identified a significant positive correlation between Fp and NADH (Figure 11A) and a significant positive correlation between NADH and GA (Figure 11B), similar to the results observed with group A cells that were not subjected to DTT. In addition, we found a significant positive correlation between the redox ratio and postmenstrual age (PMA) at sample collection (*p* = 0.039, R^2^ = 0.39, Figure 11C), which was not observed in group A cells (*p* = 0.11, R^2^ = 0.0017).

**Figure 12:**
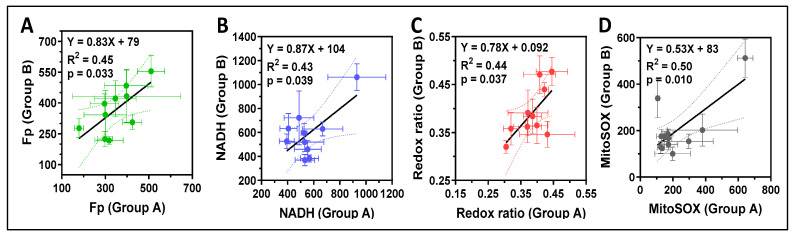
Significant positive correlations of the imaging indices between group A and group B. (**A**) Correlation of Fp; (**B**) Correlation of NADH; (**C**) Correlation of the redox ratio; (**D**) Correlation of the mitochondrial ROS. The error bars in (**A**–**D**) are standard deviations of the FOVs for any specific TA sample. All the intensity parameters (Fp, NADH, MitoSOX) are in a.u. Regression equation, goodness of fit (R^2^), and *p*-value corresponding to the regression slope are shown.

We found significant positive linear correlations for all imaging indices between the two groups (Figure 12). However, the slopes of all imaging indices were less than 1 (~10% less), especially that of mitochondrial ROS (~50% less), indicating that all the imaging indices in group B, were on average, lower than in group A. Furthermore, the intercepts also appear to be non-negligible. These findings indicate that cells in group A and group B demonstrated similar but not identical redox status and mitochondrial ROS levels.

**Figure 13:**
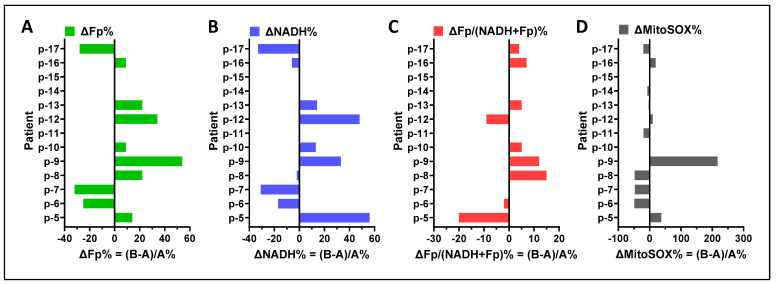
Differences in the redox status and ROS between loose (group A) and DTT-released (group B) macrophages. (**A**) Fp difference ΔFp% between two groups; (**B**) NADH difference ΔNADH% between two groups; (**C**) the redox ratio difference ΔFp/(NADH + Fp)% between two groups; (**D**) the mitochondrial ROS difference ΔROS% between two groups.

A comparison of results obtained from group A and group B cells using paired t-tests did not detect a significant difference in any of the specific imaging readouts, respectively, between cells from group A and group B. To examine the potential influence of cell preparation methods on the study results, we compared the imaging indices between group A and group B within individual subjects and found a range of positive and negative differences (Figure 13A–D). Across the study cohort, the largest individual patient differences were ~+55% in Fp and NADH readouts (in different patients), ~−20% in the redox ratio, and ~+200% in the mitochondrial ROS. The highest Fp difference corresponded to the highest ROS difference (p-9). Two out of ten cases had their Fp higher but NADH lower in group B (p-8 and p-16), but their NADH differences were negligible. The redox ratio differences for both p-6 and p-7 were also negligible and 25% of the mitochondrial ROS differences were trivial (p-10 and p-13). In summary, there does not appear to be a systematic pattern in the redox differences, indicating that the individual sample differences between the two groups of cells were unlikely due to cell preparation methods.

**Figure 14:**
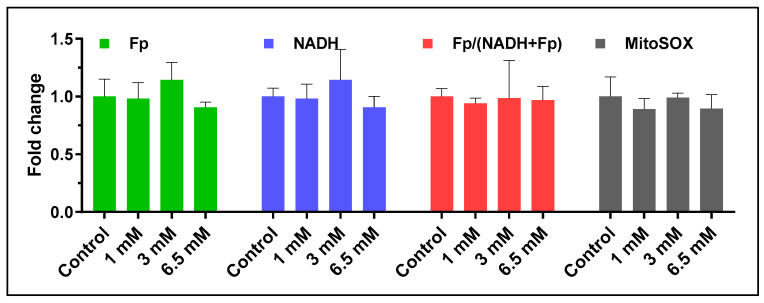
DTT effects on mouse alveolar macrophages. Treatment with DTT (1–6.5 mM) showed no significant effects on the redox indices or the mitochondrial ROS level. N = 3 FOVs, bars: mean ± SD.

We used the mouse AM cell line to confirm the absence of a DTT treatment-associated effect and support our conclusion that there were no systematic shifts in the imaging indices between group A and group B cells from infant TA samples. DTT is commonly used to release mucus-trapped cells [49,50]. It acts by conferring protection to thiol groups and reducing disulfide bonds to sulfhydryl groups in peptides and proteins. There are no available data on whether DTT affects the mitochondrial redox state. Accordingly, we treated cultured AM cell lines with DTT from 1 to 6.5 mM in the same fashion as we did for the TA samples to examine the potential effect of DTT on mitochondrial redox state over a range of plausible use concentrations. Doing so did not produce any significant effects on Fp, NADH, the redox ratio, or the mitochondrial ROS nor any trend in redox changes (Figure 14). Therefore, it is likely that the differences in the imaging indices shown in Figure 13 represent authentic cellular differences and not DTT effects.

**Figure 15:**
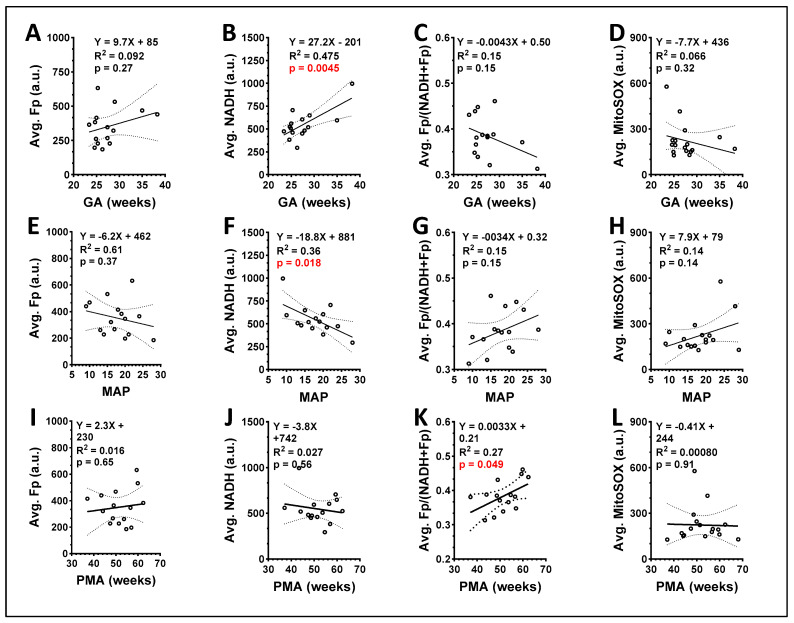
Correlations between clinical parameters and the imaging indices averaged from both loose and DTT-released macrophages with each circle representing a unique patient. (**A**–**D**) GA correlated positively with averaged NADH (*p* = 0.0045) but the other 3 redox imaging indices did not *p* > 0.05); (**E**–**H**) MAP negatively correlated with averaged NADH (*p* = 0.018) while the other redox imaging indices did not; (**I**–**L**) PMA positively correlated with averaged redox ratio (*p* = 0.049) but did not have a significant correlation with any other redox imaging indices. Regression equation, goodness of fit (R^2^), and *p*-value corresponding to the regression slope are shown, where the p values in red indicate statistically significant.

To provide single values for each study subject, we averaged the redox imaging indices between group A and B cells. We then analyzed the correlation between these averaged values and the subject’s clinical parameters. As shown in Figure 15, we found a positive correlation between GA and NADH (*p* = 0.0045, Figure 15B) and GA did not correlate with any of the other three redox imaging indices (*p* > 0.05) (Figure 15A,C,D). Furthermore, we found that ventilator MAP negatively correlated with averaged NADH (*p* = 0.018, Figure 15F) but did not correlate with the other redox imaging indices (*p* > 0.05) (Figure 15E,G,H). Lastly, we found that PMA positively correlated with the averaged redox ratio (*p* = 0.049, Figure 15K), but not with Fp, NADH, or mitochondrial ROS (Figure 15I,J,L). We did not identify any significant correlation between the imaging indices and FiO_2_ or RSS recorded at the time of TA collection.

It is potentially noteworthy that in both TA cell preparation groups, the samples from p-13, who had the highest GA (38.3 weeks), demonstrated the highest NADH and lowest redox ratio; whereas the TA sample from p-17 who had the lowest GA (23.4 weeks) had the highest mitochondrial ROS level.

## 4. Discussion

The development of novel biomarkers for disease prediction, phenotype classification, and therapy selection is an ongoing and important pursuit in neonatal medicine. In infants, the collection and analysis of TA fluid provides an important, real-time window into the ongoing physiologic state of the lung. Examination of unstained TA specimens using microscopy has the advantage of requiring fewer cells and minimal sample preparation relative to other analytical techniques. This approach has been used to obtain rapid histological information on lung fluid obtained from premature infants and may provide an early and reliable indicator of developmental change characteristics of BPD [51]. In comparison, by sequentially acquiring images of white light and the intrinsic fluorescence of NADH and Fp of TA samples, the ORI microscopy technique provides not only histological information but also metabolic/physiological information on the cellular health of macrophages within the lung, as shown in our previous animal studies using alveolar macrophages in bronchoalveolar lavage [35,36]. We conducted the present study to understand the feasibility of employing ORI microscopy to examine TA macrophages collected from ventilator-dependent infants, most of whom were born extremely premature and diagnosed with grade 3 (highest severity) BPD.

We first used the mouse AM as the model system to examine ORI responses to two factors of interest: oxidative stress (a hallmark of BPD pathophysiology) and caffeine (a prominent drug used to reduce BPD risk during neonatal intensive care). We observed a negative linear correlation between NADH level and H_2_O_2_ concentration. Additionally, we observed a positive linear correlation between Fp level and H_2_O_2_ concentration and between the redox ratio and H_2_O_2_ concentration. Furthermore, a more oxidized shift of the mitochondrial redox status of AM induced by exogenous hydrogen peroxide corresponded to higher mitochondrial ROS and more lipid peroxidation. These data show that the ORI technique not only provides histological information but also yields a quantitative measure of the mitochondrial redox metabolism in live alveolar macrophages and show how their redox indices respond to acute oxidative challenge. Moreover, the caffeine experiment suggests that high doses of caffeine shift mitochondria to a more oxidized metabolic state by lowering NADH levels. In our vitro model, caffeine did not offer protection against oxidative stress in lung macrophages.

These data, along with our previous findings demonstrating the sensitivity of ORI to specific genetic factors (such as surfactant protein-A2) and sex differences in mice exposed to oxidative stress [35,36] support the utility of ORI in studying ex vivo lung macrophages in infants. ORI enables the quantitative assessment of redox metabolism and oxidative injury, which is particularly valuable given the challenge of obtaining this information with biochemical assays and the importance of assessing lung macrophage health in respiratory disease. 

In our next set of experiments, we applied ORI to TA samples collected from 17 human infants and showed that the ORI technique readily detected NADH and Fp signals and enabled quantitative imaging of the mitochondrial ROS. We found that both the mitochondrial redox status and ROS levels varied among ventilator-dependent preterm infants most of whom were diagnosed with grade 3 BPD, suggesting that this imaging technique has the potential to detect heterogeneity in BPD disease states and may help identify specific disease endotypes. Notably, we did not observe a correlation between measured redox indices and mitochondrial ROS of lung macrophages obtained from TA samples, indicating that the redox indices reflect a cellular state that is independent of mitochondrial ROS.

In addition to imaging the loose TA macrophages (group A), we also evaluated the macrophages released from phlegm (group B). While cells in both groups showed similar correlations between NADH and GA and between NADH and MAP, phlegm-trapped macrophages may exhibit a different redox status than those that were not trapped for individual samples. It is possible that the microenvironment in phlegm may influence the activation state, functionality, cytokine production, and oxidative stress of macrophages, all of which can affect their mitochondrial redox state and ROS levels. Further method confirmation studies are needed to verify these findings.

Gestational age at birth is the strongest perinatal predictor of BPD and its severity [52]. In this cohort of 17 patients, we identified a significant positive correlation between gestational age and the observed level of NADH in TA macrophages. This novel result requires validation in a larger cohort owing to the limited number of infants born at >32 weeks’ gestation in this study. Furthermore, future studies that perform serial sampling of TA fluid from infants across different gestational and postmenstrual ages are needed to quantify potential developmental and longitudinal changes in TA macrophage redox state.

We observed a negative correlation between NADH and the administered ventilator MAP (a surrogate marker of respiratory disease severity). Intracellular NAD level is regulated by genetic factors but can also be affected by postnatal exposures. Both hyperoxia and mechanical ventilation can induce oxidative stress in lung tissue [53,54], which in turn can diminish NAD due to the activation or rapid synthesis of NAD^+^-consuming enzyme poly(ADP)-ribosyl polymerase (PARP) to repair oxidative stress-induced DNA damage [55]. Studies also showed that both cellular NAD^+^ and NADH levels decreased upon exposure to H_2_O_2_ [43,46]. The observed negative correlation between NADH and ventilator MAP may indicate consumption of NAD in response to ventilator- or oxidant-induced injury. This hypothesis is supported by our in vitro data showing a significant negative linear trend in NADH with increasing H_2_O_2_ concentration in the present study and our previous similar findings in cancer cells [33]. However, we did not detect a significant correlation between FiO_2_ and NADH levels. As such, it is unclear whether the observed finding resulted from an oxidized redox shift (i.e., increased NAD^+^ and decreased NADH) yielding a lower NADH or whether the lower NADH level may indicate a lower NAD pool/homeostasis (i.e., NADH + NAD^+^) among the critically ill infants in this cohort.

NAD^+^ together with its reduced form NADH is an essential redox co-enzyme that regulates energy metabolism including glycolysis, the tricarboxylic acid cycle, fatty acid β-oxidation, and synthesis of fatty acids. A lower NAD pool can negatively affect cellular energy metabolism. Meanwhile, NAD^+^ is also consumed by three classes of enzymes: sirtuin family deacetylases (SIRT1–7), poly(ADP)-ribosyl polymerases (PARP1–2), and cADP-ribose synthases (CD38 and CD157) [56,57]. SIRT1 has been found closely associated with BPD via a complex signaling pathway network that regulates mitochondrial biogenesis, inflammation, and response to oxidative stress, among other things [58]. Since each deacetylation of SIRT1 requires one NAD^+^ molecule, the availability of NAD^+^ closely modulates SIRT1 activity [59]. Furthermore, SIRT1 protein levels are significantly lower in BPD vs. non-BPD cases in TA leukocytes [60] and PBMC [61]. Lastly, SIRT1 is involved in the regulation of macrophage pro-inflammatory (M1)/anti-inflammatory (M2) polarization in many diseases although the mechanism in BPD remains to be elucidated.

Another source of NAD^+^ consumption is the phosphorylation of NAD^+^ to NADP^+^. NADP^+^ and its reduced form, NADPH, act as an important redox pair that is critically involved in maintaining cellular redox balance and supporting the biosynthesis of fatty acids and nucleic acids. Low NADH levels observed in the present study may reflect low NADPH because both contribute to the blue fluorescence signals detected by ORI (but NADH contribution is usually much more than NADPH). NADPH can be depleted by ROS [62]. A lower NADPH is indicative of a lower ratio of reduced glutathione (GSH) to oxidized glutathione (GSSG), i.e., the GSH/GSSG ratio, which is an indicator of cellular health [63]. As a free radical scavenger and an inhibitor of lipid peroxidation, GSH is critical for protecting cells from oxidative stress [64]. Some evidence has linked the sex specificity in oxidative stress to glutathione metabolism with males having a weaker antioxidant defense compared to females in the neonatal period, indicating the possible involvement of estrogen which promotes the activation of glutathione metabolism [65]. Future studies should examine similar sex-based differences in TA samples obtained from infants with BPD.

Furthermore, NADPH is preferred over NADH as a substrate for the NADPH oxidase (NOX) family of ROS-generating enzymes [66], which can be an important source of oxidative stress [67]. Several NOX isoforms (e.g., NOX1, NOX2, NOX4) have been found expressed and can be activated in lung macrophages [67,68,69], where NOX4 resides in the mitochondria [70,71] and also regulates mitochondrial ROS production [72]. It has also been reported that inflammatory cytokines can induce NOX-dependent ROS production in macrophages in mice [73]. There is a growing body of support for the involvement of NOX enzyme(s) in the development and/or progression of BPD [74,75]. Future studies can examine the possible involvement of NOX in lung macrophage model systems by incorporating general (e.g., diphenyleneiodonium) and isoform-specific NOX inhibitors, such as GSK2795039 which specifically inhibits NOX2, and GKT137831, which specifically inhibits NOX1 and NOX4.

In addition to their regulating role in redox and metabolism, NAD^+^/NADH and NADP^+^/NADPH play an essential role in functional morphodynamic behavior of macrophages, such as phagocytotic capacity, where cytosolic NAD^+^ was shown to regulate morphofunction by directly coupling to local, actin-based, cytoskeletal dynamics [76]. Thus, NAD^+^/NADH and NADP^+^/NADPH are essential for maintaining a large array of biological processes [56].

BPD is a complex disease with heterogeneous phenotypes. Many advanced analytical tools have been employed to study BPD, including cellular flow cytometry [77], omics [53,78], single-cell RNA sequencing [79], etc. Although our initial analysis focused on the global averaging method to obtain the mean redox measures for each patient, the redox status of individual cells can also be determined as shown in our previous works [41,80], which may provide important insights for future single-cell analysis.

Our study has several limitations. Firstly, our ORI technique cannot detect NAD^+^ directly. To directly confirm NAD deficiency in TA macrophages, biochemical assays such as enzymatic NAD cycling assay can be employed. Alternatively, methods capable of simultaneously measuring NAD^+^ and NADH within a single cell in a single run can be utilized [46]. Secondly, the present study had only 17 patient samples and limited clinical data. Our results support the plausibility of a relationship between lower NADH, and basic clinical characteristics associated with disease risk in newborns. However, larger studies with serial biosampling and robust clinical data collection are needed to build on this proof-of-concept demonstration and properly assess the utility of ORI of lung macrophages in disease characterization in high-risk newborns.

## 5. Conclusions

The present study demonstrated the feasibility of employing optical redox imaging microscopy to study tracheal aspirates obtained from ventilator-dependent infants. To our knowledge, this is the first study to report on the application of the ORI microscopy technique in this population and to reveal possible correlative relationships between clinical parameters and intracellular NADH levels in TA macrophages. This study has established a framework for utilizing ORI for future investigation of BPD and other infant lung diseases using TA specimens. The study supports our hypothesis that clinically and pathologically important heterogeneity can be observed in ORI measures in infants who have or are at risk for developing BPD. Future work will be carried out by enrolling extremely preterm infants at different stages of BPD development to determine the association between ORI measures of TA macrophages and physiological measures of lung disease and BPD phenotype.

## Figures and Tables

**Table 1:** Clinical parameters of the patients.

ID	Gestational Age (GA, Weeks)	Postmenstrual Age at Sample Collection (PMA, Weeks)	FiO_2_ (%) *	Ventilator Mean Airway Pressure (MAP)	RSS **
p-1	26.3	54.7	30	28	8.4
p-2	35.0	49.9	21	10	2.1
p-3	24.6	57.0	48	20	9.6
p-4	27.9	47.4	25	14	3.5
p-5	28.7	44.1	45	16	7.2
p-6	24.9	53.7	38	13	4.9
p-7	27.4	48.6	36	17	6.1
p-8	24.7	62.4	32	19	6.1
p-9	25.3	51.4	64	21	13.4
p-10	25.0	37.1	45	18	8.1
p-11	27.7	44.4	35	17	6.0
p-12	27.4	56.6	79	20	15.8
p-13	38.3	43.4	24	9	2.2
p-14	28.4	68.1	100	29	29.0
p-15	25.3	59.4	26	22	5.7
p-16	29.0	59.9	24	15	3.6
p-17	23.4	49.0	58	24	13.9

* Fraction of inspired oxygen; ** respiratory severity score = FiO_2_ × MAP—higher score is a proxy for more severe lung disease.

## Data Availability

Source data files containing numerical data used to generate the graphical displays will be available for sharing upon request and agreement.

## References

[1] Osterman M.J.K., Hamilton B.E., Martin J.A., Driscoll A.K., Valenzuela C.P. (2023). Births: Final Data for 2021.

[2] Jensen E.A., Edwards E.M., Greenberg L.T., Soll R.F., Ehret D.E.Y., Horbar J.D. (2021). Severity of Bronchopulmonary Dysplasia Among Very Preterm Infants in the United States. Pediatrics.

[3] Jensen E.A., Schmidt B. (2014). Epidemiology of bronchopulmonary dysplasia. Birth Defects Res. A Clin. Mol. Teratol..

[4] Thébaud B., Goss K.N., Laughon M., Whitsett J.A., Abman S.H., Steinhorn R.H., Aschner J.L., Davis P.G., McGrath-Morrow S.A., Soll R.F. (2019). Bronchopulmonary dysplasia. Nat. Rev. Dis. Prim..

[5] Jensen E.A., Dysart K., Gantz M.G., McDonald S., Bamat N.A., Keszler M., Kirpalani H., Laughon M.M., Poindexter B.B., Duncan A.F. (2019). The Diagnosis of Bronchopulmonary Dysplasia in Very Preterm Infants. An Evidence-based Approach. Am. J. Respir. Crit. Care Med..

[6] U.S. Burden of Disease Collaborators (2013). The State of US Health, 1990–2010: Burden of Diseases, Injuries, and Risk Factors. JAMA.

[7] Humayun J., Löfqvist C., Ley D., Hellström A., Gyllensten H. (2021). Systematic review of the healthcare cost of bronchopulmonary dysplasia. BMJ Open.

[8] Sillers L., Alexiou S., Jensen E.A. (2020). Lifelong pulmonary sequelae of bronchopulmonary dysplasia. Curr. Opin. Pediatr..

[9] Stoll B.J., Hansen N.I., Bell E.F., Walsh M.C., Carlo W.A., Shankaran S., Laptook A.R., Sánchez P.J., Van Meurs K.P., Wyckoff M. (2015). Trends in Care Practices, Morbidity, and Mortality of Extremely Preterm Neonates, 1993-2012. Jama.

[10] Lee S.M., Sie L., Liu J., Profit J., Lee H.C. (2021). Evaluation of trends in Bronchopulmonary Dysplasia and Respiratory Support Practice for Very Low Birth Weight Infants: A Population-Based Cohort Study. J. Pediatr..

[11] Siddaiah R., Oji-Mmuo C.N., Montes D.T., Fuentes N., Spear D., Donnelly A., Silveyra P. (2021). MicroRNA Signatures Associated with Bronchopulmonary Dysplasia Severity in Tracheal Aspirates of Preterm Infants. Biomedicines.

[12] Heydarian M., Schulz C., Stoeger T., Hilgendorff A. (2022). Association of immune cell recruitment and BPD development. Mol. Cell Pediatr..

[13] Yue L., Lu X., Dennery P.A., Yao H. (2021). Metabolic dysregulation in bronchopulmonary dysplasia: Implications for identification of biomarkers and therapeutic approaches. Redox Biol..

[14] Milan A., Priante E., Nardo D., Tosato F., Pantano G., Baraldi E., Zaramella P. (2017). Early Macrophage Activation in Preterm Newborns and Respiratory Disease. J. Child. Sci..

[15] Sahoo D., Zaramela L.S., Hernandez G.E., Mai U., Taheri S., Dang D., Stouch A.N., Medal R.M., McCoy A.M., Aschner J.L. (2020). Transcriptional profiling of lung macrophages identifies a predictive signature for inflammatory lung disease in preterm infants. Commun. Biol..

[16] Clement A., Chadelat K., Sardet A., Grimfeld A., Tournier G. (1988). Alveolar macrophage status in bronchopulmonary dysplasia. Pediatr. Res..

[17] Aghai Z.H., Kode A., Saslow J.G., Nakhla T., Farhath S., Stahl G.E., Eydelman R., Strande L., Leone P., Rahman I. (2007). Azithromycin Suppresses Activation of Nuclear Factor-kappa B and Synthesis of Pro-inflammatory Cytokines in Tracheal Aspirate Cells From Premature Infants. Pediatr. Res..

[18] Booth G.R., Al-Hosni M., Ali A., Keenan W.J. (2009). The utility of tracheal aspirate cultures in the immediate neonatal period. J. Perinatol..

[19] Xue J., Schmidt S.V., Sander J., Draffehn A., Krebs W., Quester I., De Nardo D., Gohel T.D., Emde M., Schmidleithner L. (2014). Transcriptome-based network analysis reveals a spectrum model of human macrophage activation. Immunity.

[20] Johnston L.K., Rims C.R., Gill S.E., McGuire J.K., Manicone A.M. (2012). Pulmonary macrophage subpopulations in the induction and resolution of acute lung injury. Am. J. Respir. Cell Mol. Biol..

[21] Viola A., Munari F., Sánchez-Rodríguez R., Scolaro T., Castegna A. (2019). The Metabolic Signature of Macrophage Responses. Front. Immunol..

[22] O’Neill L.A., Pearce E.J. (2016). Immunometabolism governs dendritic cell and macrophage function. J. Exp. Med..

[23] Ryan D.G., O’Neill L.A.J. (2020). Krebs Cycle Reborn in Macrophage Immunometabolism. Annu. Rev. Immunol..

[24] Michaeloudes C., Bhavsar P.K., Mumby S., Xu B., Hui C.K.M., Chung K.F., Adcock I.M. (2020). Role of Metabolic Reprogramming in Pulmonary Innate Immunity and Its Impact on Lung Diseases. J. Innate Immun..

[25] Thapa B., Lee K. (2019). Metabolic influence on macrophage polarization and pathogenesis. BMB Rep..

[26] Langston P.K., Shibata M., Horng T. (2017). Metabolism Supports Macrophage Activation. Front. Immunol..

[27] Miskolci V., Tweed K.E., Lasarev M.R., Britt E.C., Walsh A.J., Zimmerman L.J., McDougal C.E., Cronan M.R., Fan J., Sauer J.-D. (2022). In vivo fluorescence lifetime imaging of macrophage intracellular metabolism during wound responses in zebrafish. eLife.

[28] Ghukasyan V.V., Heikal A.A. (2018). Natural Biomarkers for Cellular Metabolism: Biology, Techniques, and Applications.

[29] Heikal A.A. (2010). Intracellular coenzymes as natural biomarkers for metabolic activities and mitochondrial anomalies. Biomark. Med..

[30] Xu H.N., Li L.Z. (2014). Quantitative redox imaging biomarkers for studying tissue metabolic state and its heterogeneity. J. Innov. Opt. Health Sci..

[31] Georgakoudi I., Quinn K.P. (2012). Optical imaging using endogenous contrast to assess metabolic state. Annu. Rev. Biomed. Eng..

[32] Chance B., Schoener B., Oshino R., Itshak F., Nakase Y. (1979). Oxidation-reduction ratio studies of mitochondria in freeze-trapped samples. NADH and flavoprotein fluorescence signals. J. Biol. Chem..

[33] Podsednik A., Jacob A., Li L.Z., Xu H.N. (2020). Relationship between optical redox status and reactive oxygen species in cancer cells. React. Oxyg. Species.

[34] Cortassa S., O’Rourke B., Aon M.A. (2014). Redox-Optimized ROS Balance and the relationship between mitochondrial respiration and ROS. Biochim. Biophys. Acta (BBA) Bioenerg..

[35] Xu H.N., Lin Z., Gandhi C.K., Amatya S., Wang Y., Li L.Z., Floros J. (2020). Sex and SP-A2 dependent NAD(H) Redox Alterations in Mouse Alveolar Macrophages in Response to Ozone Exposure: Potential Implications for COVID-19. Antioxidants.

[36] Xu H.N., Floros J., Li L.Z., Amatya S. (2021). Imaging NAD(H) Redox Alterations in Cryopreserved Alveolar Macrophages from Ozone-Exposed Mice and the Impact of Nutrient Starvation during Long Lag Times. Antioxidants.

[37] Zorov D.B., Juhaszova M., Sollott S.J. (2014). Mitochondrial Reactive Oxygen Species (ROS) and ROS-Induced ROS Release. Physiol. Rev..

[38] Kayton A., Timoney P., Vargo L., Perez J.A. (2018). A Review of Oxygen Physiology and Appropriate Management of Oxygen Levels in Premature Neonates. Adv. Neonatal Care.

[39] Schmidt B., Roberts R.S., Davis P., Doyle L.W., Barrington K.J., Ohlsson A., Solimano A., Tin W. (2006). Caffeine therapy for apnea of prematurity. N. Engl. J. Med..

[40] Jensen E.A. (2020). What is bronchopulmonary dysplasia and does caffeine prevent it?. Semin. Fetal Neonatal Med..

[41] Jacob A., Xu H.N., Stout A.L., Li L.Z. (2022). Subcellular analysis of nuclear and cytoplasmic redox indices differentiates breast cancer cell subtypes better than nuclear-to-cytoplasmic area ratio. J. Biomed. Opt..

[42] De la Haba C., Morros A., Martínez P., Palacio J.R. (2016). LPS-Induced Macrophage Activation and Plasma Membrane Fluidity Changes are Inhibited Under Oxidative Stress. J. Membr. Biol..

[43] Ye M., Zhao Y., Wang Y., Xie R., Tong Y., Sauer J.-D., Gong S. (2022). NAD(H)-loaded nanoparticles for efficient sepsis therapy via modulating immune and vascular homeostasis. Nat. Nanotechnol..

[44] Schraufstatter I.U., Hyslop P.A., Hinshaw D.B., Spragg R.G., Sklar L.A., Cochrane C.G. (1986). Hydrogen peroxide-induced injury of cells and its prevention by inhibitors of poly(ADP-ribose) polymerase. Proc. Natl. Acad. Sci. USA.

[45] Carson D.A., Seto S., Wasson D.B. (1986). Lymphocyte dysfunction after DNA damage by toxic oxygen species. A model of immunodeficiency. J. Exp. Med..

[46] Xie W., Xu A., Yeung E.S. (2009). Determination of NAD+ and NADH in a Single Cell under Hydrogen Peroxide Stress by Capillary Electrophoresis. Anal. Chem..

[47] Bartolome F., Abramov A.Y. (2015). Measurement of mitochondrial NADH and FAD autofluorescence in live cells. Methods Mol. Biol..

[48] Xu H.N., Feng M., Nath K., Nelson D., Roman J., Zhao H., Lin Z., Glickson J., Li L.Z. (2019). Optical redox imaging of lonidamine treatment response of melanoma cells and xenografts. Mol. Imaging Biol..

[49] Pignatti P., Delmastro M., Perfetti L., Bossi A., Balestrino A., Di Stefano A., Pisati P., Balbi B., Moscato G. (2002). Is dithiothreitol affecting cells and soluble mediators during sputum processing? A modified methodology to process sputum. J. Allergy Clin. Immunol..

[50] Spanevello A., Migliori G.B., Sharara A., Ballardini L., Bridge P., Pisati P., Neri M., Ind P.W. (1997). Induced sputum to assess airway inflammation: A study of reproducibility. Clin. Exp. Allergy.

[51] Jacobson W., Morley C.J., South M. (1992). Microscopic observations on tracheal aspirates from ventilated neonates. II. The onset of bronchopulmonary dysplasia and other changes. Eur. J. Pediatr..

[52] Greenberg R.G., McDonald S.A., Laughon M.M., Tanaka D., Jensen E., Van Meurs K., Eichenwald E., Brumbaugh J.E., Duncan A., Walsh M. (2022). Online clinical tool to estimate risk of bronchopulmonary dysplasia in extremely preterm infants. Arch. Dis. Child. Fetal Neonatal Ed..

[53] Capasso L., Vento G., Loddo C., Tirone C., Iavarone F., Raimondi F., Dani C., Fanos V. (2019). Oxidative Stress and Bronchopulmonary Dysplasia: Evidences From Microbiomics, Metabolomics, and Proteomics. Front. Pediatr..

[54] Perrone S., Tataranno M.L., Buonocore G. (2012). Oxidative stress and bronchopulmonary dysplasia. J. Clin. Neonatol..

[55] Bouchard V.J., Rouleau M., Poirier G.G. (2003). PARP-1, a determinant of cell survival in response to DNA damage. Exp. Hematol..

[56] Xiao W., Wang R.-S., Handy D.E., Loscalzo J. (2017). NAD(H) and NADP(H) Redox Couples and Cellular Energy Metabolism. Antioxid. Redox Signal..

[57] Blacker T.S., Duchen M.R. (2016). Investigating mitochondrial redox state using NADH and NADPH autofluorescence. Free Radic. Biol. Med..

[58] Yang K., Dong W. (2021). SIRT1-Related Signaling Pathways and Their Association With Bronchopulmonary Dysplasia. Front. Med..

[59] Qiu Y., Zhou X., Liu Y., Tan S., Li Y. (2021). The Role of Sirtuin-1 in Immune Response and Systemic Lupus Erythematosus. Front. Immunol..

[60] Mody K., Saslow J.G., Kathiravan S., Eydelman R., Bhat V., Stahl G.E., Pyon K., Bhandari V., Aghai Z.H. (2012). Sirtuin1 in tracheal aspirate leukocytes: Possible role in the development of bronchopulmonary dysplasia in premature infants. J. Matern. Fetal Neonatal Med..

[61] Tan F., Dong W., Lei X., Liu X., Li Q., Kang L., Zhao S., Zhang C. (2018). Attenuated SUMOylation of sirtuin 1 in premature neonates with bronchopulmonary dysplasia. Mol. Med. Rep..

[62] Pollak N., Niere M., Ziegler M. (2007). NAD Kinase Levels Control the NADPH Concentration in Human Cells*. J. Biol. Chem..

[63] Owen J.B., Butterfield D.A. (2010). Measurement of oxidized/reduced glutathione ratio. Methods Mol. Biol..

[64] Espinosa-Diez C., Miguel V., Mennerich D., Kietzmann T., Sánchez-Pérez P., Cadenas S., Lamas S. (2015). Antioxidant responses and cellular adjustments to oxidative stress. Redox Biol..

[65] Lavoie J.-C., Tremblay A. (2018). Sex-Specificity of Oxidative Stress in Newborns Leading to a Personalized Antioxidant Nutritive Strategy. Antioxidans.

[66] Vermot A., Petit-Härtlein I., Smith S.M.E., Fieschi F. (2021). NADPH Oxidases (NOX): An Overview from Discovery, Molecular Mechanisms to Physiology and Pathology. Antioxidants.

[67] Bedard K., Krause K.-H. (2007). The NOX Family of ROS-Generating NADPH Oxidases: Physiology and Pathophysiology. Physiol. Rev..

[68] Carnesecchi S., Deffert C., Pagano A., Garrido-Urbani S., Métrailler-Ruchonnet I., Schäppi M., Donati Y., Matthay M.A., Krause K.-H., Argiroffo C.B. (2009). NADPH Oxidase-1 Plays a Crucial Role in Hyperoxia-induced Acute Lung Injury in Mice. Am. J. Respir. Crit. Care Med..

[69] Carvalho C.G., Procianoy R.S., Neto E.C., Silveira R.C. (2018). Preterm Neonates with Respiratory Distress Syndrome: Ventilator-Induced Lung Injury and Oxidative Stress. J. Immunol. Res..

[70] Shanmugasundaram K., Nayak B.K., Friedrichs W.E., Kaushik D., Rodriguez R., Block K. (2017). NOX4 functions as a mitochondrial energetic sensor coupling cancer metabolic reprogramming to drug resistance. Nat. Commun..

[71] Larson-Casey J.L., Gu L., Kang J., Dhyani A., Carter A.B. (2021). NOX4 regulates macrophage apoptosis resistance to induce fibrotic progression. J. Biol. Chem..

[72] He C., Larson-Casey J.L., Davis D., Hanumanthu V.S., Longhini A.L.F., Thannickal V.J., Gu L., Carter A.B. (2019). NOX4 modulates macrophage phenotype and mitochondrial biogenesis in asbestosis. JCI Insight.

[73] Bhattacharya S., Idol R.A., Yang W., Rojas Márquez J.D., Li Y., Huang G., Beatty W.L., Atkinson J.J., Brumell J.H., Bagaitkar J. (2022). Macrophage NOX2 NADPH oxidase maintains alveolar homeostasis in mice. Blood.

[74] Menden H.L., Xia S., Mabry S.M., Navarro A., Nyp M.F., Sampath V. (2016). Nicotinamide Adenine Dinucleotide Phosphate Oxidase 2 Regulates LPS-Induced Inflammation and Alveolar Remodeling in the Developing Lung. Am. J. Respir. Cell Mol. Biol..

[75] Kimble A., Robbins M.E., Perez M. (2022). Pathogenesis of Bronchopulmonary Dysplasia: Role of Oxidative Stress from ‘Omics’ Studies. Antioxidants.

[76] Venter G., Oerlemans F.T.J.J., Willemse M., Wijers M., Fransen J.A.M., Wieringa B. (2014). NAMPT-Mediated Salvage Synthesis of NAD+ Controls Morphofunctional Changes of Macrophages. PLoS ONE.

[77] Zaramella P., Munari F., Stocchero M., Molon B., Nardo D., Priante E., Tosato F., Bonadies L., Viola A., Baraldi E. (2019). Innate immunity ascertained from blood and tracheal aspirates of preterm newborn provides new clues for assessing bronchopulmonary dysplasia. PLoS ONE.

[78] Piersigilli F., Lam T.T., Vernocchi P., Quagliariello A., Putignani L., Aghai Z.H., Bhandari V. (2019). Identification of new biomarkers of bronchopulmonary dysplasia using metabolomics. Metabolomics.

[79] Hurskainen M., Mižíková I., Cook D.P., Andersson N., Cyr-Depauw C., Lesage F., Helle E., Renesme L., Jankov R.P., Heikinheimo M. (2021). Single cell transcriptomic analysis of murine lung development on hyperoxia-induced damage. Nat. Commun..

[80] Xu H.N., Jacob A., Li L.Z. (2022). Optical Redox Imaging Is Responsive to TGFβ Receptor Signalling in Triple-Negative Breast Cancer Cells. Adv. Exp. Med. Biol..

