# Correction: Xu et al. Use of Optical Redox Imaging to Quantify Alveolar Macrophage Redox State in Infants: Proof of Concept Experiments in a Murine Model and Human Tracheal Aspirates Samples. *Antioxidants* 2024, *13*, 546

**DOI:** 10.3390/antiox13101262

**Published:** 2024-10-18

**Authors:** He N. Xu, Diego Gonzalves, Jonathan H. Hoffman, Joseph A. Baur, Lin Z. Li, Erik A. Jensen

**Affiliations:** 1 Britton Chance Laboratory of Redox Imaging, Department of Radiology, Perelman School of Medicine, University of Pennsylvania, Philadelphia, PA 19104, USA; jhh286@cornell.edu (J.H.H.); linli@pennmedicine.upenn.edu (L.Z.L.); 2 Department of Surgery, Perelman School of Medicine, University of Pennsylvania, Philadelphia, PA 19104, USA; dgonzalv@sas.upenn.edu; 3 Department of Physiology, and Institute for Diabetes, Obesity, and Metabolism, Perelman School of Medicine, University of Pennsylvania, Philadelphia, PA 19104, USA; baur@pennmedicine.upenn.edu; 4 Department of Pediatrics, Children’s Hospital of Philadelphia and Perelman School of Medicine, University of Pennsylvania, Philadelphia, PA 19104, USA; jensene@chop.edu

## Error in Figure

In the original publication [1], there was a mistake in “Figure 3” as published. “The unit of H_2_O_2_ concentration in the x-axis label has a typo: (µM) should be (mM)”. The corrected “Figure 3” appears below. The authors state that the scientific conclusions are unaffected. This correction was approved by the Academic Editor. The original publication has also been updated.


